# The Acute Abdomen

**Published:** 1909-12-04

**Authors:** 


### Surgery.

### THE ACUTE ABDOMEN.

Certain conditions demand immediate operation, for in that lies the only chance of saving the patient's life in diffuse peritonitis or acute intestinal obstruction due to a mechanical cause. The recognition of diffuse peritonitis is an easy matter if the patient is seen in the final stage, when the typical syndrome of physical signs is presented. But it should be remembered that the earlier stages are far from being easy of diagnosis, and a false sense of security may thus be induced, leading to a delay, short enough in itself but yet sufficiently long to diminish seriously or even destroy the patient's chances of recovery.

It is possible to divide the symptoms of acute diffuse peritonitis into three stages: (1) The stage of onset, accompanied by phenomena which we are accustomed to associate with shock—that is to say, the sudden onset of acute abdominal pain which may be referred to one particular spot, but is equally often said by the patient to be "all over the abdomen." The temperature is subnormal and the pulse weak and flabby, generally quickened, but sometimes slower than usual. Usually the patient vomits once, a reflex effect of the shock to the peritoneum. If the abdomen be examined at this moment no abnormal physical signs will be found, *i.e.* there will be no resistance nor rigidity, and the abdominal respiratory movements will be seen to be normally performed. There follows in an hour or two the stage of reaction or "latent period." This is most misleading, because in it the preceding symptoms disappear and the patient displays an apparent improvement that is nothing short of marvellous. The pain is diminished or gone, and the pulse and temperature have returned to normal. Such temporary improvement should be regarded with the gravest suspicion. In the course of an hour or two, if the patient be watched, the symptoms of the third stage, that of genuine peritonitis, will supervene. The phenomena of this are so well known as to demand only passing reference. The anxious expression, with eyes which are beginning to assume a sunken appearance, a gradually rising temperature, and a pulse which is quick and wiry, are prominent features. The position of the patient is also characteristic—he lies on his back, with his legs drawn up to relax the muscles and fascia of his abdominal wall, and he often turns his head from side to side. A patient with peritonitis never throws himself about or rolls from side to side. This is a small point but an important one, because there may be a difficulty in determining whether a patient has peritonitis or colic. A patient with colic is always restless and tries every position in turn to obtain a temporary relief from his sufferings. But the final and most convincing evidence of peritonitis is the lack of abdominal respiratory movement and *rigidity*. Both these phenomena point to one fact—that the parietal peritoneum is inflamed, and the patient's one desire is to keep it absolutely still, since the slightest movement accentuates the burning intra-abdominal pain of which the patient complains.

It sometimes occurs in hospital practice that one is called to see a patient who has had a sudden attack of abdominal pain, and has already had medical treatment, which has afforded him relief. This may be taken to indicate that he has been given morphia, especially if the pupils are much contracted, and one must always be on the look out for this, since it masks the symptoms completely and renders an accurate diagnosis impossible. If no history can be obtained as to the condition before the morphia was given, there is no alternative but to wait until its effect has worn off and then to examine the patient again. The routine practice of giving morphia to a patient who has sudden severe abdominal pain cannot be too strongly condemned: but there are, of course, occasions in which its administration is justifiable—as, for example, in the case of a patient who is seen in a country district where no facilities for performing laparotomy are at hand. In such a case morphia may be given to lessen the patient's discomfort while he is moved to the nearest hospital; but an accurate note of the symptoms and signs should be made, with a statement of the amount of morphia given, and these should be sent with the patient, if he is to be transferred to the care of a medical man.

Acute diffuse peritonitis is usually found in connection with a rupture of some viscus, or the spread of inflammation from it, *e.g.* the stomach, intestine, gall-bladder, appendix, or, in the case of the female, the genital apparatus. It follows that the past medical history will be most useful in localising the seat of the trouble; but we are concerned here rather with the recognition of the condition than with the differential diagnosis of its several causes.

Peritonitis in its final stages, in which the intestine becomes paralysed and distended, may simulate acute intestinal obstruction due to mechanical causes, *e.g.* obstruction by band. In both the onset is associated with shock, accompanied by vomiting. But in acute obstruction there is absolute constipation from the start, the vomiting becomes stercoraceous, the temperature rarely rises above the normal throughout, and there is rarely rigidity of the abdominal wall. But it must be remembered that acutely obstructed gut may give way and peritonitis supervene, so that the two conditions may be coincident.

In the earlier stages it may be mistaken for the various forms of colic. But, as has been already said, a patient with colic is apt to fling himself about, and he rarely presents true rigidity of the abdomen. An examination should, however, always be made for signs of lead poisoning.

Tabetic crises are a not uncommon feature, and a gastric crisis has before now been mistaken for a perforated gastric ulcer. Systematic examination of the pupil reactions and of the condition of the knee-jerks should, therefore, not be neglected.

